# VEO-IBD NOX1 variant highlights a structural region essential for NOX/DUOX catalytic activity

**DOI:** 10.1016/j.redox.2023.102905

**Published:** 2023-09-27

**Authors:** Josie Ward, Suisheng Zhang, Adam Sikora, Radoslaw Michalski, Yuting Yin, Aurora D'Alessio, Rachel M. McLoughlin, Vincent Jaquet, Franck Fieschi, Ulla G. Knaus

**Affiliations:** a School of Medicine, Conway Institute, University College Dublin, Dublin, Ireland; b Faculty of Chemistry, Institute of Applied Radiation Chemistry, Lodz University of Technology, Lodz, Poland; c Host-Pathogen Interactions Group, School of Biochemistry and Immunology, Trinity Biomedical Sciences Institute, Trinity College Dublin, Dublin, Ireland; d Department of Pathology and Immunology and READS Unit, Faculty of Medicine, University of Geneva, Geneva, Switzerland; e Univ. Grenoble Alpes, CNRS, CEA, UMR5075, Institut de Biologie Structurale, Grenoble, France; f Institut Universitaire de France (IUF), Paris, France

**Keywords:** NADPH oxidase, NOX, DUOX, NOX1 model, Peroxynitrite, Inflammatory bowel disease, VEO-IBD, Germ-free mice

## Abstract

Inflammatory bowel diseases (IBD) are chronic intestinal disorders that result from an inappropriate inflammatory response to the microbiota in genetically susceptible individuals, often triggered by environmental stressors. Part of this response is the persistent inflammation and tissue injury associated with deficiency or excess of reactive oxygen species (ROS). The NADPH oxidase NOX1 is highly expressed in the intestinal epithelium, and inactivating NOX1 missense mutations are considered a risk factor for developing very early onset IBD. Albeit NOX1 has been linked to wound healing and host defence, many questions remain about its role in intestinal homeostasis and acute inflammatory conditions. Here, we used *in vivo* imaging in combination with inhibitor studies and germ-free conditions to conclusively identify NOX1 as essential superoxide generator for microbiota-dependent peroxynitrite production in homeostasis and during early endotoxemia. *NOX1* loss-of-function variants cannot support peroxynitrite production, suggesting that the gut barrier is persistently weakened in these patients. One of the loss-of-function *NOX1* variants, *NOX1* p. Asn122His, features replacement of an asparagine residue located in a highly conserved HxxxHxxN motif. Modelling the NOX1-p22^phox^ complex revealed near the distal heme an internal pocket restricted by His119 and Asn122 that is part of the oxygen reduction site. Functional studies in several human NADPH oxidases show that substitution of asparagine with amino acids with larger side chains is not tolerated, while smaller side chains can support catalytic activity. Thus, we identified a previously unrecognized structural feature required for the electron transfer mechanism in human NADPH oxidases.

## Highlights

• NOX1 is crucial for microbiota and LPS-dependent intestinal ONOO^−^/ONOOH generation.
• Identification of a catalytically essential HxxxHxxN motif in NOX/DUOX.
• NOX1-p22^phox^ Asn122 modelling supports an internal oxygen capture site.

## Graphical abstract

**Figure:**
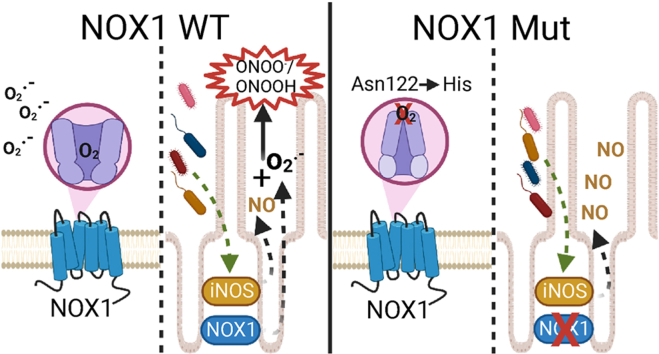

## 1 Introduction

Very early onset (VEO) inflammatory bowel disease (IBD) constitutes a pediatric IBD subgroup with rising incidence that is driven by strong genetic influence and includes genetic variants associated with primary immunodeficiency. The clinical course of VEO-IBD is often severe with pancolitis and bloody diarrhea, and many patients require early immunosuppressive treatment. Of the over 70 genes currently linked to VEO-IBD many are considered causative monogenic variants, while others are classified as risk factors [1,2]. The mechanisms involved in VEO-IBD pathogenesis are categorized into epithelial barrier defects, impaired mucosal and innate immune defense, defects in host-microbiota interaction and bacterial sensing, B and T cells defects and hyper/autoinflammation. Host defense is intimately linked to the generation of reactive oxygen species (ROS) by phagocyte NOX2 and the epithelial barrier NADPH oxidases NOX1 and DUOX2 [[3], [4], [5]]. We reported the first inactivating *NOX1* and *DUOX2* variants in VEO-IBD in 2015 [6], followed by additional variants identified by us and others [[7], [8], [9], [10], [11]]. Although many of these variants occur *de novo*, some were of Mendelian inheritance leading to disease in hemizygous males (X-linked *NOX1*) or are compound heterozygous variants (*DUOX2*). The remaining NOX1 activity of male VEO-IBD patients harboring the missense mutations *NOX1* p. N122H and *NOX1* p. T497A, or the stop-gain mutation *NOX1* p.54R>* is likely negligible or absent [8,10]. How abolished NOX1 activity triggers VEO-IBD is still not resolved as unchallenged Nox1 deficient mice display no overt phenotype in environmentally controlled conditions (SPF FELASA standard). Possible scenarios include altered Toll receptor signaling through NOX1-generated superoxide, changes in microbiota composition and host-microbiota interactions, decreased antivirulence capacity or impaired wound healing [3,12]. VEO-IBD patients expressing catalytically inactive, full-length *NOX1* presented with pancolitis, Paneth cell metaplasia, cryptitis, and crypt abscess formation; their serum LPS levels or microbiota composition were not reported [8]. In NOX1 deficiency the communication between the epithelial barrier and the microbiota is likely compromised, which may lead to dysbiosis and chronic intestinal inflammation. In mice compensatory mechanisms exist as evident by comparing the phenotype of single NOX1-3 knockout mice to triple NOX inactivated mice (*Cyba*^*nmf333*^) [13]. Here, we determined the NOX1 response to microbiota and LPS, a pathogen-associated molecular pattern (PAMP) produced by overgrowth of IBD-associated proteobacteria. Cell-based assays recapitulated the main NOX1 derived reactive product in the murine intestine, and identified, based on the VEO-IBD *NOX1* p. N122H variant, the importance of an H*xxxHxxN motif in NOX/DUOX. This structural feature is essential for the electron transfer mechanism from the distal heme to molecular O_2_, and thus for catalytic activity across all mammalian NADPH oxidases.

## 2 Materials and methods

### 2.1 Reagents

MPO inhibitor AZD5904, iNOS inhibitor 1400W (MedChemExpress); mitochondria-targeted antioxidant MitoQ (Merck); NOS inhibitor l-NAME, flavoenzyme inhibitor diphenyleneiodonium chloride (DPI), lipopolysaccharide *E. coli* O111:B4, phorbol 12-myristate 13-acetate (PMA), luminol (all Sigma-Aldrich). L-012 (Fujifilm Wako Chemicals), Hanks’ Balanced Salt solution and phosphate-buffered saline with/without calcium and magnesium (Gibco). The boronate derivative of fluorescein, FlBA, was synthesized as described previously ([14]).

### 2.2 Mice

Female and male mice used were C57BL/6J (JAX000664), B6.129X1-*Nox1*^*tm1Kkr*^/J (*Nox1*^*-/Y*^) (JAX018787) [15], B6.129S-*Cybb*^*tm1Din*^/J (*Cybb*^*-/y*^) (JAX:002365) [16], B6.*-Cyba*^*tm1819Arte*^ *(Cyba*^*−/−*^) [17], SPF and germ-free C57BL/6NTac mice. Internal *Nox1*^*+/Y*^ control mice were created by crossing *Nox1*^*-/Y*^ mice with C57BL/6J mice. All animal experiments followed the EU Directive 2010/63/EU and were approved by the Animal Research Ethics Committee and the Health Products Regulatory Authority of Ireland.

### 2.3 IVIS analysis

Mice were anesthetized with 5% isoflurane and i. p. Injected with L-012 (20 mg/kg) before 10min of whole body kinetic imaging, with 5sec or 2min exposures using the IVIS® Spectrum CT (PerkinElmer). After euthanasia intestines were removed and imaged for a further 5 s. Total flux was calculated as photons/sec. LPS (10 mg/kg) or PBS control was administered i. p. 6 h before imaging, either alone or in combination with 1400W (20 mg/kg, PBS), MitoQ (8 mg/kg, DMSO 1:1000), l-NAME (50 mg/kg, H_2_O), or AZD5904 (40 mg/kg, DMSO 1:5).

### 2.4 Molecular biology

CGW bicistronic lentiviral expression vectors with Mnd promoter were used [18]. p22^phox^ was fused at the C-terminus via alanine linker to mCerulean. Plasmids for transient transfections were pcDNA3.1 or pJ3H. Mutations were introduced using site-directed mutagenesis and verified by sequencing.

### 2.5 Quantitative real-time PCR

RNA was extracted from the murine terminal ileum using RNeasy Mini Kit (Qiagen) and reverse transcribed using High-Capacity cDNA reverse transcription kit (Thermo-Fisher). Real-time PCR was conducted using Taqman Fast Universal PCR mastermix and Taqman Gene Expression Assays (Applied Biosystems). Gene expression levels were normalized to *Gapdh* or *Hprt*, and data was analyzed by 2−ΔΔCt method. Taqman probes were *Cybb* Mm01287743_m1, *Duox2* Mm00470560_m1, *Mpo* Mm01298424_m1, *Nos2* Mm00440485_m1, *Nox1* Mm00549170_m1, *Tlr4* Mm00445273_m1, *Gapdh* Mm99999915_g1, *Hprt* Mm03024075_m1 (Applied Biosystems).

### 2.6 Cell lines and transfection

Differentiation to M0 bone marrow-derived macrophages (BMDMs) was for 6 days in DMEM, 10% FBS, M-CSF (20 ng/ml, Peprotech). Polarization to M1 BMDMs was with LPS (100 ng)/IFNγ (10 ng/ml) for 24 h. CHO cells stably expressing NOXO1 and NOXA1 [19] were stepwise transduced with p22^phox^-mCerulean and NOX1. CHO-NOXO1-NOXA1-p22^phox^ cells were transiently transfected with iNOS (pCMV-NOS2) and respective NOX1 WT or mutant as indicated. CHO-NOXO1-NOXA1-p22^phox^ cells and COS7-p22^phox^ cells [20] were transfected with Lipofectamine 3000 (Invitrogen), H661-DUOXA2 cells [18] with Fugene (Promega).

### 2.7 Immunoblotting

Primary antibodies used were NOX1 [21]; NOX2 (53/gp91^phox^, BD); V5-tag (V5-10), Vinculin (hVIN-1), β-Tubulin (ONS.1A6), F-Actin (polyclonal) (all Sigma-Aldrich); HA-tag (16B12, Covance); p22^phox^ (FL-195, Santa Cruz); p47^phox^ and p67^phox^ [22].

### 2.8 Flow cytometry

Unpermeabilized cells were stained for NOX4 (clone 20.3) [20] or anti-HA (DUOX2) for 20min, followed by FITC-conjugated anti-rabbit secondary antibody (30min; Invitrogen), fixation, and analysis using a CytoFLEX LX cytometer.

### 2.9 RONS measurements

ROS production was assessed with/without PMA stimulation (500 ng/ml for NOX2, 100 ng/ml for NOX1) using luminol-based chemiluminescence as described in Ref. [22]. The percentage RLU/WT was calculated at the peak. H_2_O_2_ was determined via HVA assay [20]. DUOX2 was stimulated with PMA (100 ng/ml) and thapsigargin (1 μM) and the H_2_O_2_ concentration was determined (nmol H_2_O_2_/mg protein). iNOS expressing cells were pre-incubated with/without l-NAME (10 μM) for 30min in suspension, followed by adding l-arginine (12 mM) and FIBA (10 μM) and stimulation with PMA (100 ng/ml). 5 × 10^5^ cells in 200μl/well were measured at Ex495/Em519 for 30min using a Synergy MX plate reader.

### 2.10 Griess reaction

The modified Griess reaction [23] was performed on 100 μl of cell supernatant after 1 h stimulation with PMA (100 ng/ml). The absorbance was read at 520–550 nm after overnight incubation, standard curves for nitrate were interpolated.

### 2.11 Modelling NOX1/p22^phox^ heterodimer

Nox1 structural model was generated by AlphaFold server [24]. The p22^phox^ structure used for the heterodimer NOX1/p22^phox^ model building came from the cryo-EM solved structure of the NOX2-p22^phox^ core complex [25]. Structural analysis, representation as well as mutation modelling were done using the PyMOL Molecular Graphics System, Version 2.5.4 Schrödinger, LLC.

## 3 Results

### 3.1 NOX1 is essential for intestinal peroxynitrite generation

**Fig. 1:**
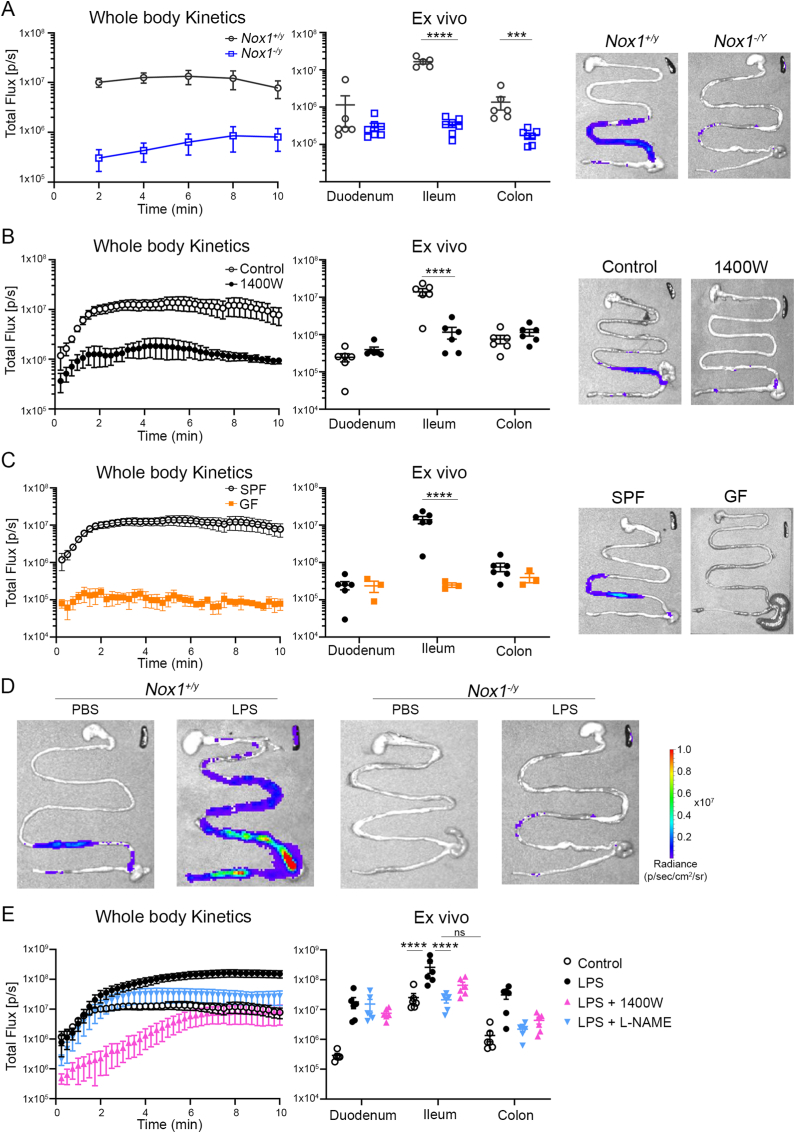
**Peroxynitrite formation in mice is dependent on NOX1, iNOS, and the microbiota.** Mice were injected with L-012 immediately before IVIS whole body or *ex vivo* imaging. Kinetic curves, regional analysis of intestinal flux, and representative images of excised intestines are shown. (A) *Nox1*^*+/Y*^ and *Nox1*^*-/Y*^ mice at homeostasis (n = 6/group). (B) Wildtype mice injected with 1400W or PBS 6 h prior to imaging (n = 5–6/group). (C) Comparison of SPF WT mice with GF WT mice (n = 3–6/group). (D) *Nox1*^*+/Y*^ and *Nox1*^*-/Y*^ mice were injected with LPS or PBS 6 h prior to imaging (n = 5–6/group). (E) WT mice were injected with LPS, LPS and NOS inhibitors as indicated, or PBS 6 h prior to imaging (n = 5–6/group). A two-way ANOVA with multiple comparisons test was performed on normalized data. (non-significant (NS) P > 0.05, ***P ≤ 0.005, ****P ≤ 0.0001).

We reported that intestinal ROS generation visualized by L-012 luminescence in unchallenged C57BL/6 mice was predominantly dependent on NOX1 with minor contribution of other NOX isoforms [13]. Whole-body kinetics were followed up by *ex vivo* analysis of excised mouse intestines, indicating that the majority of the L-012 signal was localized to the terminal ileum (Fig. 1A). The intestines of *Nox1*^*-/y*^ mice emitted only minimal luminescence in ileum and colon, confirming the dominant role of NOX1 in providing ileal ROS in homeostasis. Loss of the ileal L-012 signal in Nox1-deficient mice was even more remarkable as other NADPH oxidases such as *Cybb* (NOX2) and *Duox2*, and inducible nitric oxidase synthetase 2 (*Nos2*; iNOS) were upregulated in *Nox1*^*-/y*^ mice (Fig. S1A). *Duox2*, already highly expressed in wildtype ileum (Ct24), was significantly upregulated in NOX1-deficient ileum (Ct19), suggesting that the luminol derivative L-012 does not react with DUOX2 generated H_2_O_2_ *in vivo*. In contrast, certain reactive species such as peroxynitrite (ONOO^−^/ONOOH) and hypochlorous acid (HOCl) react rapidly with the L-012 probe *in vivo* [[26], [27], [28], [29]]. Injection of 1400W, a specific, irreversible iNOS inhibitor, resulted in loss of the intestinal L-012 signal in whole-body kinetics and *ex vivo* intestine imaging (Fig. 1B), indicating intestinal peroxynitrite generation by NOX1 and iNOS activity in homeostasis. As both NOX1 and iNOS are expressed in the intestinal barrier epithelium in mice and humans [[30], [31], [32], [33], [34]], their continuous stimulation is likely due to interaction with the microbiota. In germ-free C57BL/6 WT mice IVIS imaging after injection of L-012 did not detect any signal *in vivo* or *ex vivo* (Fig. 1C) which is attributable to a combination of decreased *Nox1*/*Nos2* expression and insufficient stimulation of NOX1 activity when microorganisms and PAMPs are completely absent (Fig. S1C).

Intestinal inflammation is accompanied by changes in the composition and diversity of the microbiota that include upregulation of gram-negative bacteria (e.g., proteobacteria) and elevated plasma LPS levels. *Nox1*^*-/y*^ and internal wildtype mice were injected with LPS or PBS prior to L-012 injection and IVIS imaging to monitor intestinal *in vivo* ONOO^−^/ONOOH generation. Whole body and *ex vivo* intestinal luminescence in wildtype mice was highly increased by LPS compared to non-treated wildtype mice (Fig. 1D, S1D), while L-012 luminescence was hardly detectable in NOX1 knockout mice. Inhibitor studies showed that iNOS was involved in LPS-induced intestinal peroxynitrite generation in ileum and colon, while the duodenal luminescence signal was not decreased by NOS inhibitors (Fig. 1E). A contribution of HOCl generated by innate immune cell NOX2-derived O_2_^•-^/H_2_O_2_ and myeloperoxidase (MPO) activity or the involvement of mitochondrial ROS was excluded (Fig. S1E). LPS induced the upregulation of *Nox1, Nos2, Duox2* and *Mpo* in murine ileum (Figs. S1A and B), indicating upregulation and/or *de novo* expression of NADPH oxidases and iNOS, as well as neutrophil infiltration. NOX1 and iNOS are not only expressed in intestinal epithelial cells but also in proinflammatory M1 macrophages [35]. To exclude any influence of NOX1 deficiency on iNOS upregulation in macrophages, bone marrow derived macrophages (BMDMs) were prepared from *Nox1*^*-/y*^, *Cybb*^*-/y*^ and *Cyba*^*−/−*^ mice (M0) and then polarized with LPS/IFNγ to M1 BMDMs. No differences were observed in *Nos2* or iNOS expression when NOX isoforms were inactivated (Fig. S2A). Other flavoenzymes potentially affecting *Nos2*/iNOS upregulation were excluded by treating M0 BMDM with diphenyleneiodonium (DPI) during the M0→M1 transition (Fig. S2B). To rule out any contribution of NOX2-derived superoxide in intestinal peroxynitrite generation *Cybb*^*-/y*^ mice were treated with LPS or PBS as described above for *Nox1*^*-/y*^ mice. No significant change in whole body or *ex vivo* total flux was detected (Fig. S2C), indicating that NOX1 activity is essential for homeostatic and LPS-induced ONOO^−^/ONOOH generation in the murine intestine.

### 3.2 VEO-IBD *NOX1* variant cannot generate peroxynitrite

**Fig. 2:**
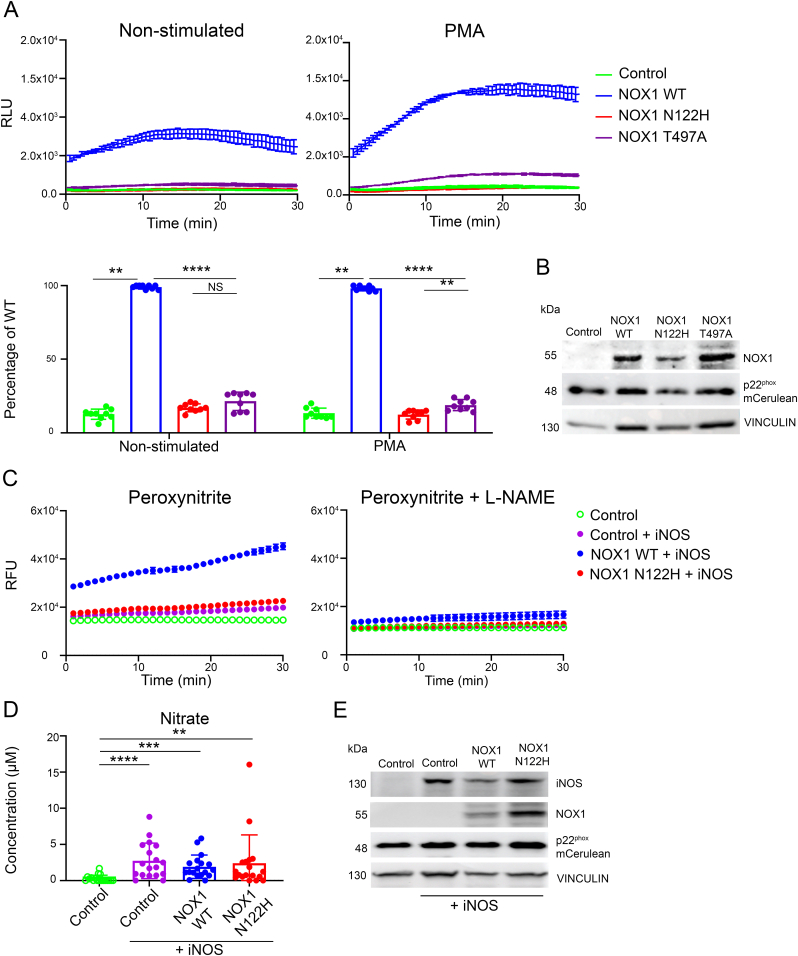
**VEO-IBD NOX1 mutations abolish catalytic activity and prevent peroxynitrite formation.** (A) Superoxide generation of CHO-NOXO1-NOXA1-p22^phox^ cells (control) or cells co-expressing NOX1 WT, NOX1 N122H or NOX1 T497A in the presence or absence of PMA (in RLU and as % of WT). (B) Immunoblot of cell lines in (A). (C) Detection of peroxynitrite with FIBA in the absence or presence of 10 μM l-NAME (in RFU). Cell lines in (A) co-expressing iNOS as indicated were stimulated with PMA. (D) Nitrate quantification in supernatants of indicated cell lines after PMA stimulation (1 h). (E) Immunoblot of cell lines in (C). A two-way ANOVA with multiple comparisons test was performed (A) or a Kruskal-Wallis test with multiple comparisons (D). (non-significant (NS) P > 0.05, *P ≤ 0.05, **P ≤ 0.01, ***P ≤ 0.001, ****P ≤ 0.0001).

The remaining catalytic activity of loss-of-function *NOX1* variants identified in VEO-IBD patients varies [6,8]. Two male pediatric patients carried NOX1 mutants with significantly decreased L-012 luminescence when analyzed in transiently transfected HCT116 cells [8]. To characterize these mutants in more detail, we prepared stable CHO cell lines expressing the NOX1 heterodimerization partner p22^phox^, the NOX1 complex components NOXO1 and NOXA1, and either NOX1 WT or the two patient mutants (NOX1 N122H, NOX1 T497A). NOX1 complex expressing CHO cells were produced by antibiotic selection (NOXO1, NOXA1) followed by 2-step lentiviral transduction, each time sorting for cells with medium expression levels. Luminol chemiluminescence indicated basal and PMA stimulated superoxide generation by NOX1 WT that was abolished in NOX1 N122H expressing cells, and significantly reduced in NOX1 T497A expressing cells (Fig. 2A and B). CHO-NOX1 WT and CHO-NOX1 N122H expressing cells were then transiently transfected with iNOS and analyzed for peroxynitrite generation using the ONOO^−^/ONOOH specific probe FIBA and for nitrate with the Griess reaction (Fig. 2C–E). The patient NOX1 N122H mutant was not capable of generating ONOO^−^/ONOOH.

### 3.3 Conserved NOX1 Asn122 is essential for catalytic activity of all mammalian NADPH oxidases

**Fig. 3:**
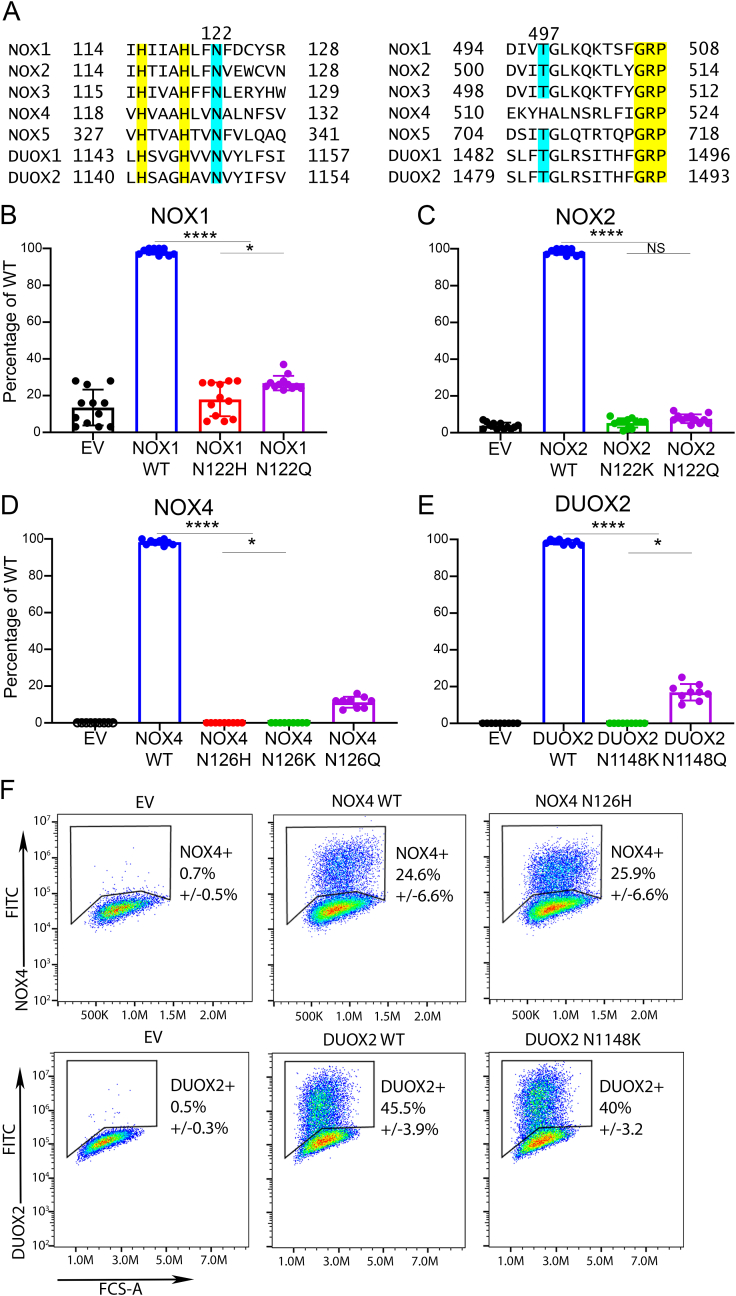
**Conserved asparagine in the HxxxHxxN motif is essential for the catalytic activity of NOX/DUOX.** (A) NOX1-5 and DUOX1-2 sequence alignments with asparagine (blue) and histidines (yellow), and with threonine (blue) and GRP sequence (yellow). (B) CHO-NOXO1-NOXA1-p22^phox^ cell lines were transfected with NOX1 WT and mutants, followed by PMA stimulation. (C) COS7-p22^phox^ cells were transfected with p47^phox^, p67^phox^, and NOX2 WT or mutants, followed by PMA stimulation. (D) COS7-p22^phox^ cells were transfected with NOX4 WT or mutants, constitutive H_2_O_2_ generation was measured. (E) H661-DUOXA2 cells were transfected with DUOX2 or mutants, PMA/thapsigargin stimulated H_2_O_2_ generation was measured. (F) Percentage of NOX4 or DUOX2 positive cells by cell surface staining, transfections as above. A one-way ANOVA with multiple comparisons (NOX1,2) or Kruskal-Wallis test with multiple comparisons (NOX4, DUOX2) was performed (non-significant (NS) P > 0.05, *P ≤ 0.05, ****P ≤ 0.0001). (For interpretation of the references to colour in this figure legend, the reader is referred to the Web version of this article.)

Sequence analysis of NOX1 patient variants indicates conservation of Asn122 (NOX1/NOX2 position) in an H*xxxHxx**N** motif across all 7 mammalian NADPH oxidases, while threonine (NOX1 T497) is also highly conserved but absent in NOX4 (Fig. 3A). Asn122 is located upstream of His115(H*) that coordinates the outer heme and of His119 in the putative oxygen reduction center, which may indicate a crucial role of Asn122 in molecular oxygen reduction via an outer sphere mechanism [36]. To determine the importance of asparagine located at this position in the NOX domain, point mutations of Asn to His/Gln/Lys residues were introduced into NOX1, NOX2, NOX4 and DUOX2. Glutamine was chosen for overall similarity to the original asparagine residue, and lysine as positively charged residue comparable to the histidine replacement in the NOX1 mutant. The full set of the respective NOX or DUOX complexes were reconstituted in cells and assayed for superoxide or H_2_O_2_ generation. All three asparagine substitutions did not support catalytic activity of oxidases (Fig. 3B–E, Figs. S3A–B) with only the Asn to Gln substitution being capable of producing minimal H_2_O_2_ (10–15%) by NOX4 and DUOX2 complexes. Cell surface localization of NOX4 and DUOX2 WT and mutants was comparable, indicating that mutation of Asn in the H*xxxHxx**N** motif affected only the catalytic activity but not protein expression, membrane complex heterodimerization or subcellular localization of oxidases (Fig. 3F, Figs. S3C–F).

### 3.4 Structural analysis of the modeled NOX1-p22^phox^ complex

**Fig. 4:**
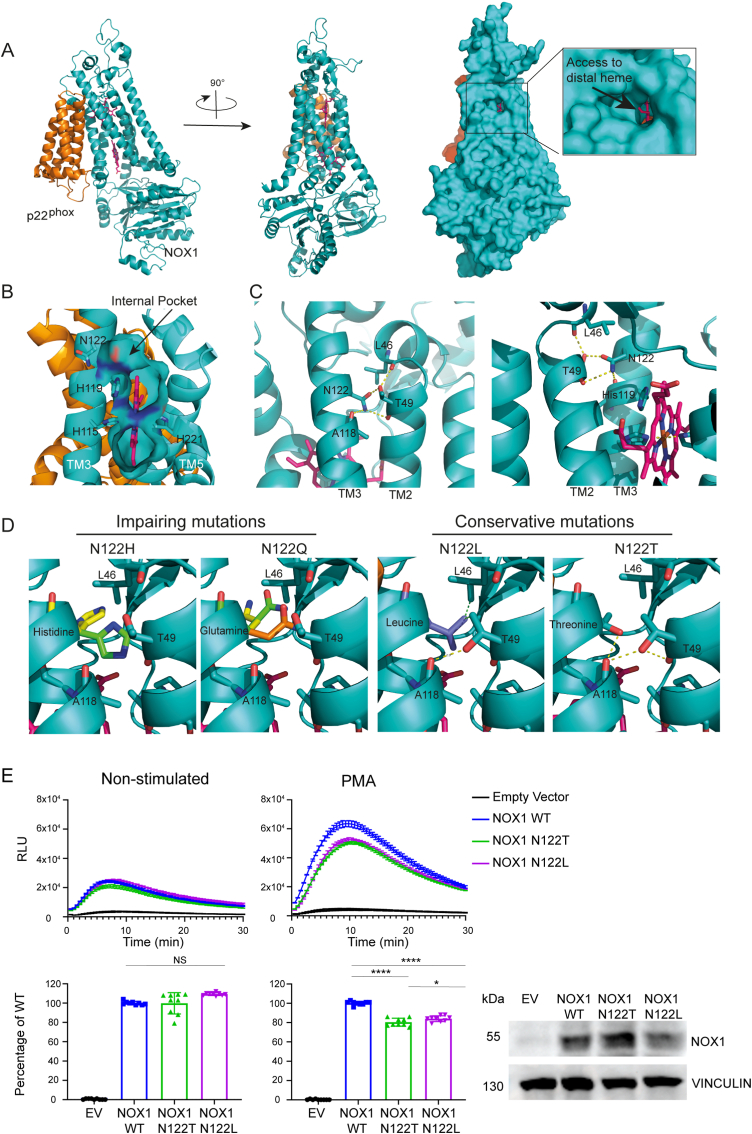
**NOX1 modelling reveals structural features involved in the oxygen reduction center.** (A) Cartoon representation of a NOX1/p22^phox^ heterodimer model inside view (left) or eclipsed (p22^phox^ behind, center). On the right, eclipsed view in surface mode revealing lateral access to the distal heme (zoom), NOX1 (teal blue), p22^phox^ (orange). (B) Zoom, on a vertical section within NOX1, on the distal heme region in NOX1 with cavities highlighted in surface mode. An additional internal pocket at the distal heme site is revealed. The side chains of the heme-chelating histidines as well as N122 and H119, delimiting the internal pocket, are represented by sticks. The vertical section within NOX1 allows to see only TM3, TM4 and partly TM5, while the others have been eliminated from front for clarity. (C) Zoom in NOX1 structure of the N122 environment at the interface between TM3 and TM2 observed from the outside (left) or the inside of NOX1 (right). (D) Modelling the structural impact of mutations at position N122. Modification performed by the use of the mutagenesis function within Pymol software. From left to right, the N122H, N122Q, N122L and N122T mutations. For the first two, several possible rotamers have been represented simultaneously for the corresponding mutated side chain. In the case of N122L and N122T modeled mutations another rotamer of the facing T49 side chain has been considered as possible adaptation to the mutation of N122. In (C) and (D), dotted yellow line represented H-bond and dotted green line possible hydrophobic interaction. (E) CHO-NOXO1-NOXA1-p22^phox^ cells were transfected with NOX1 WT and mutants, followed by PMA stimulation. Immunoblots indicate equal protein expression. One-way ANOVA with repeated measures (non-significant (NS) P > 0.05, *P ≤ 0.05, ****P ≤ 0.0001). (For interpretation of the references to colour in this figure legend, the reader is referred to the Web version of this article.)

The cryo-EM structure of the NOX2-p22^phox^ complex has recently been solved [25,37]. AlphaFold 2 was used to model NOX1-p22^phox^ using the coordinates from the NOX2 structure. The cartoon representation of the NOX1/p22^phox^ heterodimer model allows to see the two proximal and distal hemes within the transmembrane domain (Fig. 4A left and center). They are not visible anymore in a surface representation mode (Fig. 4A, right), illustrating their location in the heart of the protein structure protected from the outside. However, access from the outside to the distal heme is visible and might constitute an opening to allow the substrate, molecular dioxygen, to enter and reach an internal pocket close to the heme during the catalytic process. Interestingly, this open access is located in the upper part of the transmembrane domain at the level of the external leaflet of the lipidic bilayer. These observations have also been made within the NOX2 structure (not shown). Such a path would imply that O_2_ has to diffuse within the bilayer to access its catalytic site, suggesting the same return path for the superoxide produced after reduction, since no other exit could be found. One could consider it as a surprise that the release of ROS occurs in the bilayer and not directly to the outside considering that lipids are sensitive targets for ROS. However, it is also known that lipids are mainly sensitive to hydroxyl radicals while poorly reactive towards superoxide anions. To strengthen the premise of an putative O_2_ access tunnel from the outside of the protein to the distal heme, an analysis was carried out using the program MOLEonline (https://mole.upol.cz) to search for channels and cavities in proteins [38], comparing the resting state structure of the entire NOX2 [37] with the NOX1 model used here. Assuming conserved features between NOX1 and NOX2, analysis of the latter can be performed with more confidence as it presents an experimental structure, and those results will then be extrapolated to the NOX1 model. The predictions from this tunnel search are presented in Fig. S4. Three putative tunnels can be found in the inactivated state of NOX2 - two tunnels with access to the distal heme coming from the two opposite lateral sides and one tunnel coming from the extracellular cap structure. All tunnels contain in their path an area with a constriction radius below 1.5 Å which is considered too small for O_2_ access. However, it seems likely that minimal conformational dynamics or small structural rearrangements occur upon NOX2 activation, thereby expanding one of these preexisting tunnels and enabling a functional pathway for O_2_ access to the distal heme. We postulate that the grey tunnel would require much more structural adaptation as access is blocked by p22^phox^ within the complex (Fig. S4). The two other tunnels (red and green) are fully accessible and are at least partially conserved in the NOX1 model, suggesting that these two tunnels might be more relevant. A remarkable insight from this analysis is the realization that all proposed tunnels end at an internal pocket whose lateral side is delimited by the residue N122 highlighted in this work.

Coming back to the NOX1 model, and the use of a representation mode where the cavities are represented as a surface, a section inside the model shows the internal space hosting the heme group but also additional spaces, and in particular this internal pocket isolated from the outside on the upper face of the distal heme (Fig. 4B). Again, it is interesting to note that this internal pocket is delimited by His119 but also Asn122 which is the residue mutated in the pathological VEO-IBD variant studied here. His119 is strictly conserved in mammalian NOXs but also in a prokaryotic NOX5 homologue, CsNOX5, where this histidine has been postulated to be essential for O_2_ reduction [39], stabilizing it near the distal heme. However, in CsNOX5, the postulated site for O_2_ reduction, materialized in the CsNOX5 structure by a crystallographic water molecule, was open and accessible directly from the top of the protein i.e., from the outside (not shown). Thus, this internal pocket, located at the same position, is a good candidate for the O_2_ reduction site, confining the O_2_ in a constrained space and potentiating the outer sphere mechanism of reduction. It is striking to consider that Asn122 blocks lateral access to this pocket. Asn122 in TM3 is directly involved in a network of hydrogen bonds that help stabilize the TM3/TM2 interface and extracellular loop 1. Thus, mutations of N122 could destabilize the local organization and alter the correct packing of TM2 and TM3 but could also impact the size and shape of the neighboring pocket supposed to be the site of O_2_ reduction (Fig. 4C). The modelling of the N122H mutation inactivating NOX1, initially identified in a VEO-IBD patient, suggests that the larger side chain of histidine will either create a steric clash or, precisely due to its size, will orient towards the neighboring space and fill partially the pocket (Fig. 4D, rotamer green and yellow respectively). Both issues may either disorganize or fill the O_2_ pocket and may hamper O_2_ access and thus catalytic activity. A similar observation can be made for the mutation to glutamine. Depending on the different possible rotamers, the glutamine side chain will fill the pocket or conflict with facing L46 or even T49 from TM2 (rotamer yellow, green, and orange respectively in Fig. 4D). With the lysine side chain being even larger, the same structural effects can be expected (not shown) and the resulting functional consequences of an Asn to Lys mutation are shown in Fig. 3. It cannot be ruled out that a local structural disruption due to these substitutions could propagate conformationally over a long distance in the structure, leading to other alterations such as the orientation of the hemes or their stabilization. Given the model presented here and the environment surrounding N122, with free space in its vicinity, this last option is not the one we favor.

To lend credence to the molecular hypotheses on the impact of mutations, we tried to predict additional N122 mutations that could, based on this molecular model, maintain NOX1 activity. N122L and N122T mutations have been evaluated (Fig. 4D, right panel). N122 exchange by a leucine will maintain same volume of the side chain without any clash between TM3 and TM2. To accommodate this substitution and the loss of the H-Bond network stabilization usually induced by N122, the T49 side chain from TM2 could invert its orientation, allowing new H-Bonding with A118 and generating a small stabilizing hydrophobic cluster with its methyl group, the L46 side chain and the new Leu122 mutated residue. All things considered, the N122L mutation should keep the topology and stabilization of the interface. The N122T mutation should fit as well, with a slightly smaller size, same polarity, and certain H-Bond possibility, and adding again rotation of Leu49 to maintain the stabilizing H-Bond between TM3 and TM2, with negligible effects on the global NOX1 structure and the local environment expected. To test these hypotheses NOX1 N122T and NOX1 N122L mutations were prepared and compared to NOX1 WT after transfection into CHO cells containing NOXO1, NOXA1 and p22^phox^. The expression of NOX1 wildtype and mutants, and their baseline superoxide production were comparable, while phorbol ester (PMA)-stimulated superoxide generation of mutants showed a modest decrease (15–20%) of the NOX1 WT output (Fig. 4E).

It is interesting to note that Asn122 conserved in animal NADPH oxidases is replaced by a conserved Cys residue in plant NOX homologs (RboH enzymes) [40]. Typically, a Cys residue could correspond in size to a structurally non-disturbing mutation. This H*xxxHxxN motif does not exist in prokaryotic NOX which diverge from eukaryotic sequences. These enzymes contain very small extracellular loops [41] and thus have no cap structure that could impede the access to the outer heme. There is no need to organize a pocket as the outer heme is likely always accessible from the outside as exemplified in the structure of csNOX5 [39].

We also considered structural reasons for the loss of catalytic activity of the VEO-IBD *NOX1* p. T497A mutation. T497 belongs to a sequence, uniquely inserted into the NOX DH domain, that adds a predicted ***α***-helix element to the classical fold of the FNR family of reductase domains (Fig. S5A). This sequence has been termed the NIS (NADPH oxidase insertion sequence) [42] and is thought to play a role in the regulation of NOX activity. Its modification at various positions within the NOX2 isoenzyme impacted the first electron transfer step from NADPH and/or the assembly of cytosolic factors. However, from the position of T497 in the NOX1 model and its structural environment, it is difficult to anticipate why replacement by alanine is detrimental. Alanine will not create an obvious steric disruption and T497 seems not to be critical for stabilizing interactions (Fig. S5B). Of note, this region is not visible in the available structures of NOXs, suggesting a flexible element. The importance of T497 might be revealed in the context of a fully assembled NADPH oxidase complex that may reorganize the NIS region. A shorter NIS sequence lacking the threonine is a feature of NOX4 which is constitutively active.

## 4 Conclusions

Monogenic variants in VEO-IBD are clinically important and may demand different therapy regimens [1]. Consequently, a better understanding of gene function and the pathogenicity associated with gene malfunction at a very early age is required. As the rarity of patients with monogenic variants and their frequently severe clinical presentation usually prohibits in depth functional analysis, cell/organoid-based methodologies and animal experiments provide valuable insights. Mouse models engineered to carry IBD relevant pathogenic changes rarely develop ileitis or colitis, possibly due to welfare and health standards, but reveal disease phenotypes when challenged, when closely related, compensatory genes are co-deleted, or when two risk factors are jointly altered [13,43,44]. In the case of NOX1 inactivating NOX1-3 (*Cyba*^*nmf333*^) in mice was sufficient to cause severe colitis upon challenge, mainly due to mucus barrier and host defense failure [13]. We set out to understand the physiological function of NOX1 in the intestine by using *in vivo*/*ex vivo* RONS imaging of healthy and LPS-treated mice. The chemiluminescent probe L-012 with its high luminescent yield and increased sensitivity performs well *in vivo*, but does not react directly with superoxide, and should be avoided for *in vitro* assays at 21% oxygen [45]. When used for intestinal imaging L-012 detects ONOO^−^/ONOOH in physiological conditions and after LPS stimulation, as shown by using specific inhibitors and NOX1/iNOS knockout mice (Fig. 1) [46,47]. These *in vivo* studies indicate that inducers of intestinal inflammation such as elevated LPS levels or the chemical colitogen DSS [48] trigger predominantly NOX1 generated RONS. In inflammatory conditions some input of innate immune cell NOX2 cannot be excluded due to the timing of the analysis or due to the probe not detecting other relevant species (O_2_^•-^/H_2_O_2_). However, ileal *Mpo* upregulation by LPS (Fig. S1A) or analysis at the peak of DSS-induced colitis [48] support neutrophil infiltration, usually associated with NOX2 activation.

In physiological conditions NOX1 continuously provides RONS in the murine ileum. The steady-state concentration of ileal ONOO^−^/ONOOH is likely not sufficient to act in a bactericidal manner, but may alter microbial signaling, metabolism and transcription by thiol modification and tyrosine nitration [49,50]. In wildtype mice, low levels of peroxynitrite may serve as repellent to restrict access of microorganisms to the epithelium, as the ileal barrier epithelium is only covered by a loose mucus layer but contains the highest concentration of bacteria in the small intestine. Peroxynitrite in combination with antibacterial defensins and lectins will act as an additional barrier, preventing bacterial translocation. Recent reports suggest that absence of peroxynitrite is not decisive for microbiota alterations [46,48], but constant communication of microbiota with the host epithelium was essential for expression and catalytic activity of NOX1 and iNOS. Even in germ-free conditions *Nox1* (Ct 24 → 26) and *Nos2* (Ct 20 → 23) expression would be sufficient for O_2_^•-^ and NO generation, but NOX1 catalytic activity requires microbial stimulation. Constant microbial sensing by NOX1 may not take place at the colonic epithelium as long as the dense mucus layer and the overall barrier are intact. By extrapolating our animal studies to humans, we suggest that the loss-of-function *NOX1* p. N122H and *NOX1* p. T497A variants cannot not support ONOO^−^/ONOOH production, which is critically reliant on the location of NOX1 due to the extremely fast reaction between O_2_^•-^ and NO [51]. An inherent weakness in peroxynitrite signaling for mucosal defense together with potential changes in the stem cell compartment [32,52] may drive the VEO-IBD phenotype in these patients.

Inactivating NADPH oxidase variants discovered in patients with chronic granulomatous disease and hypothyroidism have greatly enhanced our understanding of structure-function relationships in NOX2 and DUOX2 complexes [53]. With the advent of NADPH oxidase cryo-EM structures the impact of many pathogenic missense mutations can finally be explained. The NOX1 structure-function studies described in this work highlight the role of an internal pocket close to the distal heme that is delimited by N122 and H119. In addition to the high level of conservation of these residues, we found that any significant change in position 122, even a reasonable one such as a change from Asn to Gln that represents a very small increase in chain size, has drastic consequences for activity. Hence, the immediate environment of the N122 residue is very sensitive and essential for NOX/DUOX catalytic activity. Our data reinforce the hypothesis of a specific O_2_ capture site enabling reduction by the outer sphere mechanism. Thus, the study of VEO-IBD variants of NOX1 has led us towards a structural feature that is essential for the electron transfer mechanism at the final stage of the reaction, from the distal heme to molecular O_2_.

However, many questions are still not resolved including the NOX isoform specific changes in the formation of the oxygen reduction center as they likely differ between superoxide and H_2_O_2_ generating NADPH oxidases. Even with the available structures the significance of the highly conserved Asn122 (in NOX1, NOX2) for oxidase catalytic activity could only be deduced by functional assessment. For the near future mutational and functional analysis of NOX/DUOX patient variants will still be a valuable and predictive tool that will enhance the interpretation of novel NOX/DUOX structures and inform future therapeutic approaches.

## Declaration of competing interest

The authors declare the following financial interests/personal relationships which may be considered as potential competing interests:Ulla Knaus reports financial support was provided by 10.13039/501100001602Science Foundation Ireland. Adam Sikora reports financial support was provided by Polish National Science Center. Radoslaw Michalski reports financial support was provided by Polish National Science Center. 10.13039/100016417Aurora D'Alessio reports financial support was provided by Erasmus Mobility EU. Rachel McLoughlin reports financial support was provided by 10.13039/100010269Wellcome Trust. Franck Fieschi reports financial support was provided by French Universities Institute.

## Data Availability

Data will be made available on request.
