# Supplementary material for: VEO-IBD NOX1 variant highlights a structural region essential for NOX/DUOX catalytic activity
Source: Redox Biol. 2023 Sep 27;67:102905. doi: 10.1016/j.redox.2023.102905 (PMC10571032; doi:10.1016/j.redox.2023.102905)
Supplement: Multimedia component 1 [file mmc1.pdf]

SUPPLEMENTARY FIGURES 1-5

Ward et al, VEO-IBD NOX1 variant highlights a structural region essential for NOX/DUOX catalytic activity

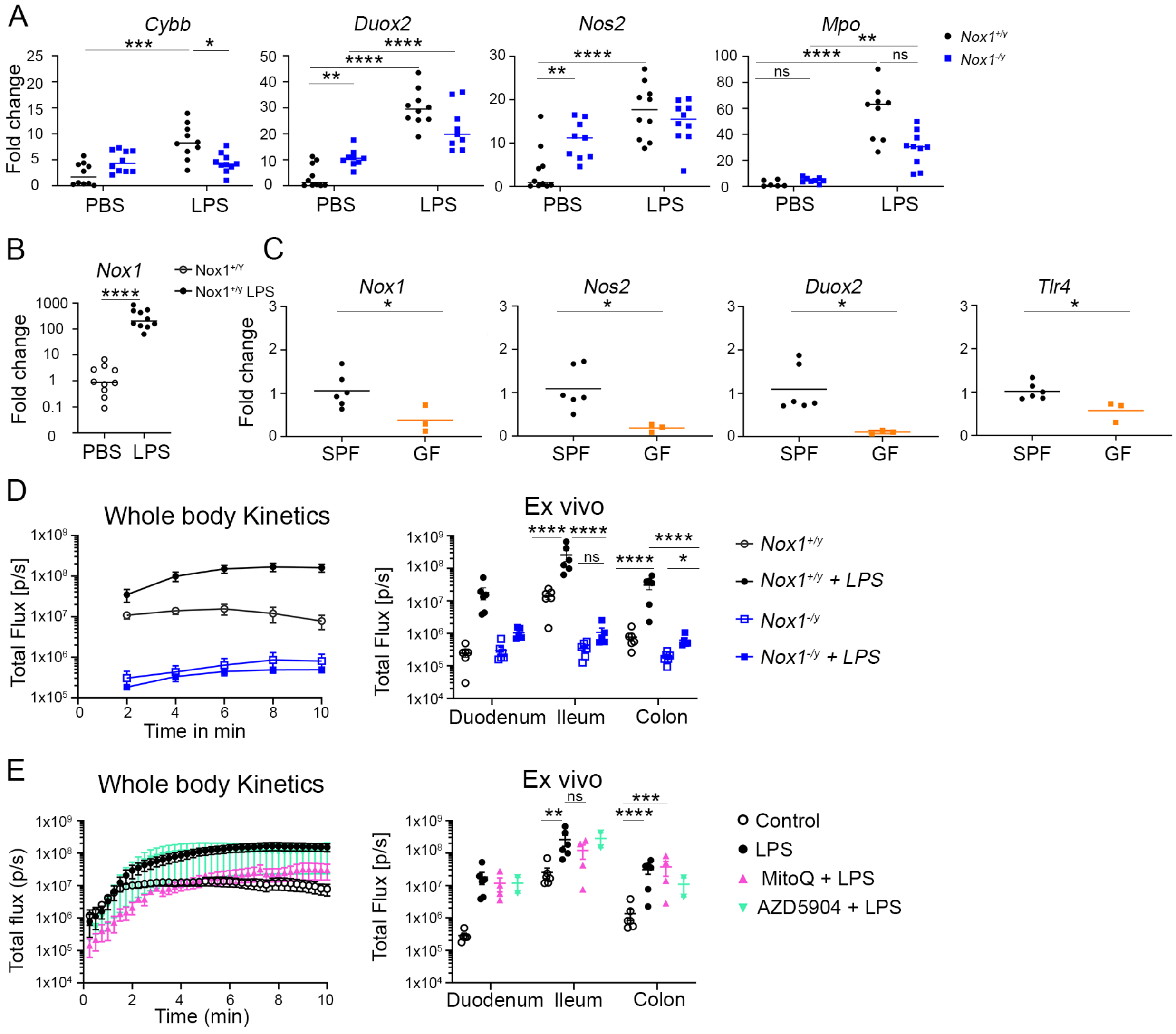

Figure S1. Gene expression and peroxynitrite generation in LPS-treated *Nox1*$^{+/Y}$ and *Nox1*$^{-/Y}$ mice, and in germ-free WT mice. (A) Ileal gene expression 6h after injection of *Nox1*$^{+/Y}$ and *Nox1*$^{-/Y}$ mice with LPS or PBS (n=6-10/group). (B) Ileal gene expression 6h after injection of *Nox1*$^{+/Y}$ mice with LPS or PBS (n=10/group). (C) Ileal gene expression of specific pathogen free (SPF) WT and germ-free (GF) WT mice (n=3-6/group). (D) *Nox1*$^{+/Y}$ and *Nox1*$^{-/Y}$ mice were injected with LPS or PBS 6h prior to imaging (n=5-6/group). Kinetic curves and regional analysis of intestinal flux are shown. (E) WT mice were injected with LPS, LPS and MitoQ, LPS and AZD5904, or PBS 6h before IVIS imaging (n=2-6/group). (A) two-way ANOVA with multiple comparisons was performed, or (B) Mann-Whitney test. C) t-test, (D, E) two-way ANOVA with multiple comparisons was performed on log-transformed data. (non-significant (NS) P > 0.05, * P ≤ 0.05), ** P ≤ 0.01), *** P ≤ 0.001), **** P ≤ 0.0001).

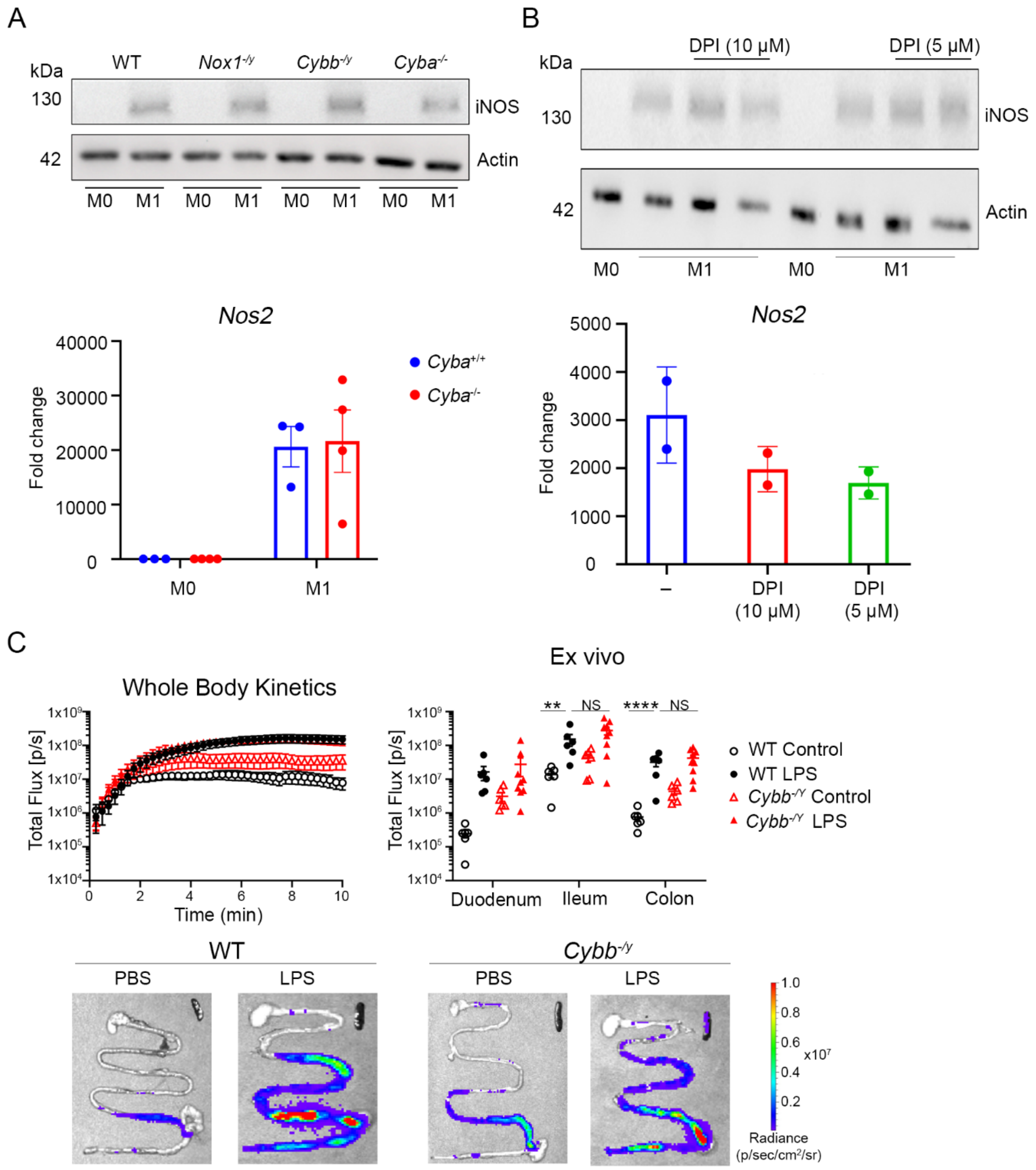

Figure S2. Upregulation of iNOS is independent of NADPH oxidase activity. iNOS immunoblot or *Nos2* qPCR of M0 or M1 BMDMs which were either (A) derived from BM of indicated mouse strains, or (B) derived from BM of WT mice and treated with/without DPI during M1 polarization (24h LPS/IFNγ). Actin served as loading control. (C) *Cybb*$^{+/Y}$ and *Cybb*$^{-/Y}$ mice were injected with LPS or PBS 6h prior to L-012 imaging (n=6-9/group). Kinetic curves, regional analysis of intestinal flux, and representative images of excised intestines are shown. Two-way ANOVA with multiple comparisons was performed on log-transformed data (non-significant (NS) P > 0.05, * P ≤ 0.05), ** P ≤ 0.01), **** P ≤ 0.0001).

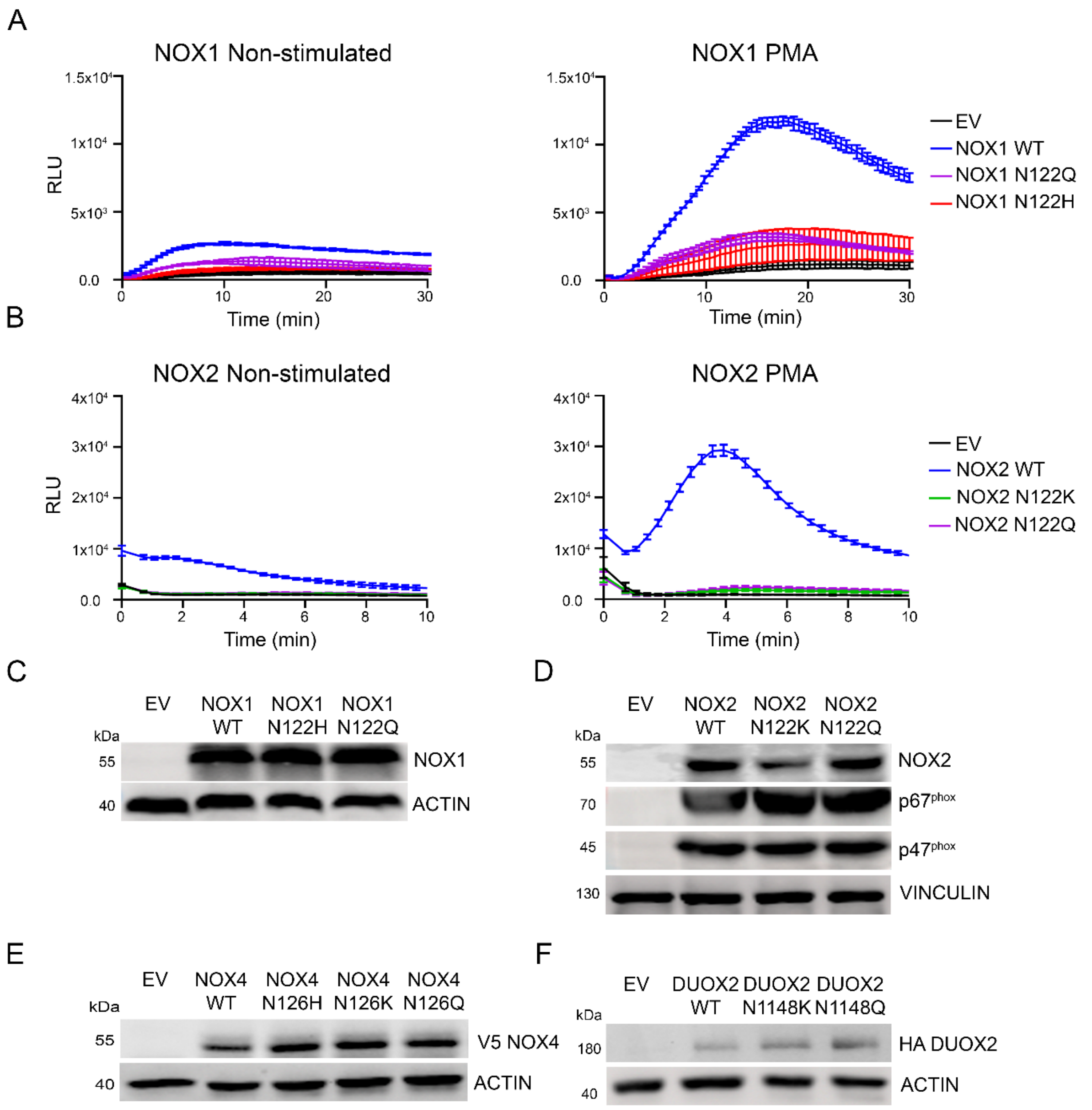

Figure S3. Superoxide generation and expression of NADPH oxidases. (A) Superoxide generation of CHO-NOXO1-NOXA1-p22^{phox} cells transfected with NOX1 WT, NOX1 N122H, NOX1 N122Q or empty vector (EV) in the presence or absence of PMA. (B) Superoxide generation of COS7-p22^{phox} cells transfected with p47^{phox}, p67^{phox}, and NOX2 WT, NOX2 N122K, NOX2 N122Q, or EV in the presence or absence of PMA stimulation. (A, B) Representative curves of luminol chemiluminescence are shown in relative luminescence units (RLU), see quantification of replicates in Figure 3B, C. (C-F) Representative immunoblots for NOX1, NOX2, NOX4 and DUOX2 expression for experiments depicted in Figure 3. Stable cell lines were used for transient transfections as indicated in Figure 3.

NOX2/p22phox with putative tunnels for O_2 diffusion

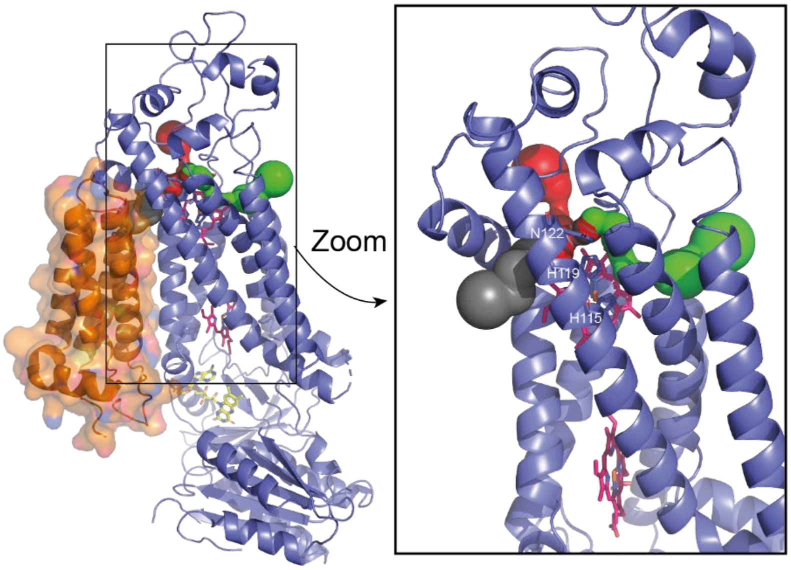

NOX1 model with putative tunnels for O_2 diffusion

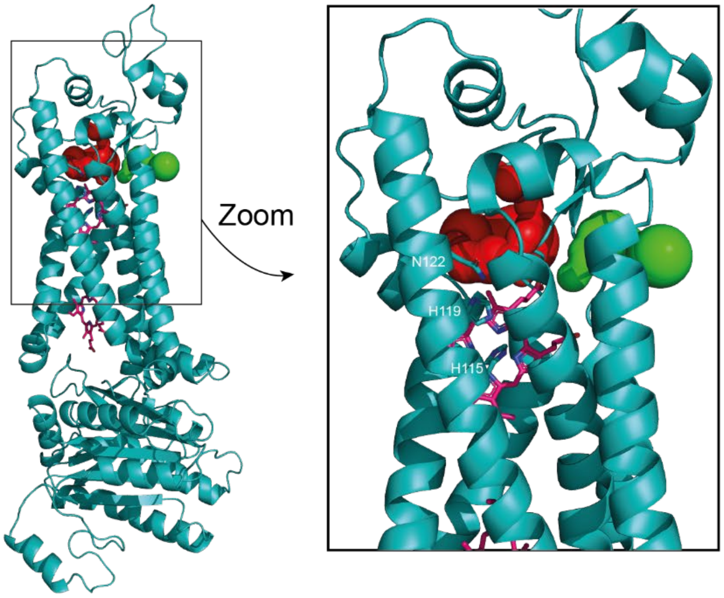

Figure S4. Tunnel search using MOLE*online* program (https://mole.upol.cz). On top, analysis performed on the NOX2 subunit (purple; PDB 8gz3; 32). Three tunnels coming from outside, in grey, red and green, are reaching an internal pocket delimitated by N122 and H119. p22^{phox} was added (left, orange) to show that it blocks access to the grey putative tunnel. Bottom, analysis performed on the NOX1 model studied in this study, identifying one tunnel in red (with two entries) sharing some path with the red NOX2 tunnel and a green tunnel, interrupted in the middle by one side chain, but also sharing some similarity with the green NOX2 tunnel. With the parameters used (default channel parameter search except for the bottleneck radius that was increased to 1.3 Å). Side chains of N122, H119 and H115 are represented in the Zoom.

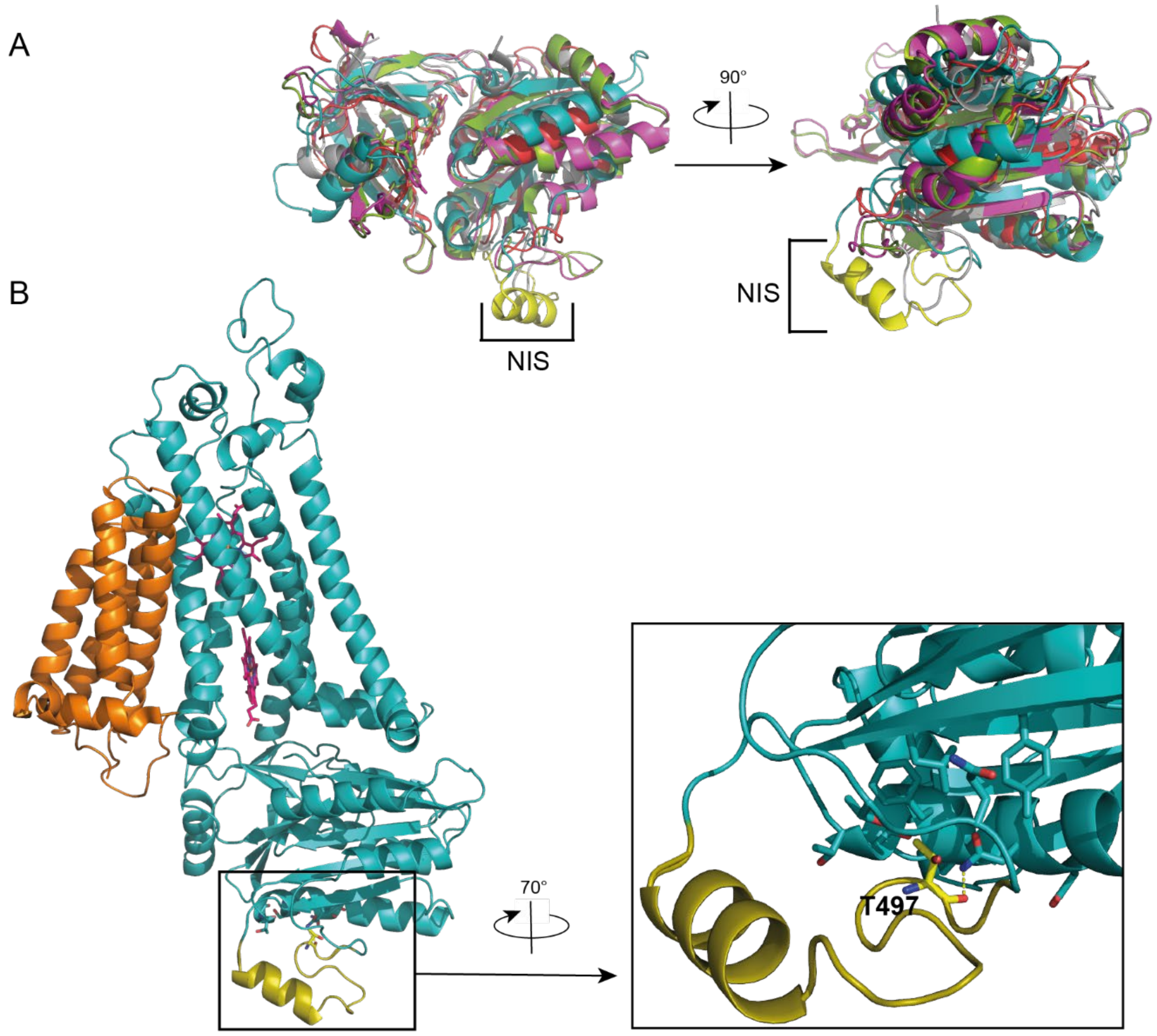

Figure S5. Structural analysis of the NOX Insertion Sequence (NIS) and NOX1 T497. A) Superimposition of reductase domains of members of the FNR family: reductase domain of Pthalate Dioxygenase reductase (2PIA, red), Pea Ferredoxin $NADP^+$ reductase (1QG0, green), Spinach Ferredoxin $NADP^+$ reductase (1FNC, magenta), *E. coli* Flavin reductase (1QFJ, grey), NOX1 Dehydrogenase domain (AlphaFold model, blue) with the NIS sequence in yellow, indicated by a black open box. B) NOX1/$p22^{phox}$ AlphaFold model. The NIS sequence is highlighted in yellow. The T497 residue is represented in stick in the zoom window.
